# Potentially traumatic experiences and mental health among asylum-seeking women of reproductive age: The significance of sexual violence and contextual risk factors

**DOI:** 10.1007/s00737-026-01697-z

**Published:** 2026-04-21

**Authors:** Satu Majlander, Tarja I. Kinnunen, Eero Lilja, Anu E. Castaneda, Natalia Skogberg, Päivikki Koponen

**Affiliations:** 1 Finnish Institute for Health and Welfare, Helsinki, Finland; 2 Tampere University, Tampere, Finland

**Keywords:** Asylum-seeker, Women, Trauma, Sexual violence, Mental health

## Abstract

**Purpose:**

Asylum-seeking women are often exposed to potentially traumatic experiences (PTEs), such as various forms of psychological and physical violence. This study examines women’s current mental health by assessing depressive and anxiety symptoms, symptoms indicating psychological trauma, and associated contextual factors.

**Methods:**

This study utilized data from the Asylum Seekers Health and Wellbeing Survey conducted in Finland in 2018. Women aged 18 to 50 years (*n* = 278) were included in the analysis and grouped by region of birth. PTEs were assessed using questions adapted from the Harvard Trauma Questionnaire. The Hopkins Symptom Checklist-25 was used to detect depressive symptoms and anxiety symptoms, and symptoms indicating psychological trauma were screened using the PROTECT Questionnaire.

**Results:**

Women who had experienced sexual violence had significantly higher odds of depressive and anxiety symptoms (OR = 6.33, 95% CI:2.86–14.05) compared to those who had not. Younger age (18–29 years) was also associated with higher odds of depressive and anxiety symptoms (OR = 2.07, 95% CI:1.10–3.89). Sexual violence (OR = 4.71, 95% CI:1.99–11.17), other PTEs (OR = 3.13, 95% CI:1.46–6.73), not having children (OR = 2.22, 95% CI:1.12–4.41) or ≥ 3 births (OR = 3.62, 95% CI:1.43–9.14), multilingualism (OR = 2.37, 95% CI:1.23–4.55), and being born in the Middle East and North Africa (OR = 2.42, 95% CI:1.19–4.95) were associated with symptoms indicating psychological trauma.

**Conclusion:**

Sexual violence and other traumatic experiences, along with contextual factors, significantly elevate the risk of mental health problems among asylum-seeking women.

**Supplementary Information:**

The online version contains supplementary material available at 10.1007/s00737-026-01697-z.

## Highlights

Sexual violence is associated with depressive and anxiety symptoms, and symptoms indicating psychological trauma in asylum-seeking women.

Other traumatic experiences and contextual factors, such as age, parity, and absence of family, are associated with mental health risks.

Trauma and contextual factors affect women’s lives and should be better recognized in research and services.

**Supplementary Information:**

The online version contains supplementary material available at 10.1007/s00737-026-01697-z.

## Introduction

An asylum seeker is an individual who has applied for international protection but whose claim has not yet been legally determined by the receiving country (IOM, 2019). Although migration tends to be more challenging for women than for men (e.g., due to limited resources and traditional caregiving responsibilities), the number of female asylum seekers continues to rise. Between 2018 and 2024, the number of asylum seekers worldwide increased from approximately 3.5 million to an estimated eight million. Women have consistently represented around 30–40% of this population. (UNHCR, The UN Refugee Agency, 2025.)

Over the past decades, women seeking asylum in Finland have often arrived from conflict-affected regions, and their countries of birth have slightly changed from year to year. In 2024, Finland received 2,399 first-time asylum applications, primarily from citizens of Somalia, Afghanistan, and Syria. Women frequently flee due to gender-based violence or persecution. (Finnish Immigration Service, 2018; 2024.)

Gender significantly shapes vulnerability during migration by influencing migration patterns (such as routes taken and travel conditions), exposure to risks, and the redefinition of social roles in new environments. Patriarchal structures, cultural norms, and socio-economic disadvantages often hinder the realization of women’s health and rights. (Wandschneider et al. 2020.) These factors disproportionately expose women to violence both in their countries before migration and throughout the migration journey (UNHCR, The UN Refugee Agency, 2025). Potentially traumatic experiences (PTEs) during migration include violence, death or separation from family members, torture, imprisonment, and witnessing harm to others, experiences common among forcibly displaced individuals regardless of where people migrate from and the countries they travel through. (Knipscheer et al. 2020.)

Limited legal pathways for migration and asylum, such as forced return policies, force individuals to rely on smugglers, increasing the dangers of migration. These conditions expose migrants to risks such as kidnapping for ransom and abuse by organized crime groups and, in some cases, State authorities. (United Nations Office on Drugs and Crime, 2018.)

The conceptual framework of this study draws on research addressing women-specific issues. It is informed by the Ottawa Charter for Health Promotion and WHO’s definitions and frameworks on health determinants and equity (World Health Organization, 1986, 2024a). Health determinants (such as living conditions, education, social status, and structural inequalities) shape health opportunities more than genetics or healthcare access, creating significant inequities. WHO emphasizes gender-specific factors, particularly those related to sexual and reproductive health and rights. Grounded in a trauma-informed, gender-sensitive approach, this study highlights how gender-based violence and forced migration profoundly affect women’s mental health (Jolof et al. 2024). Previous research indicates that asylum-seeking women frequently experience severe traumatic events that negatively impact their mental health. Both armed conflicts and the asylum process expose women to high a risk of violence. (Acharai et al. 2023; Davaki 2021; Gordon et al. 2023; Jobe 2020; Koning et al. 2021.) Women from conflict-affected or socioeconomically unstable regions are particularly vulnerable to threats against their sexual and reproductive health and rights (SRHR) (Hedström and Herder 2023). Many women flee severe human rights violations, including sexual torture, rape, and other forms of sexual and gender-based violence (SGBV), which is among the most harmful types of violence. SGBV encompasses physical, psychological, sexual, socio-economic abuse, and harmful cultural practices, and includes acts causing harm or suffering, threats, coercion, and deprivation of liberty. (UNHCR, The UN Refugee Agency, 2025.) Women who have been physically or sexually abused are more likely to experience unintended pregnancies, gynecological morbidity, miscarriages, unsafe abortions, pregnancy complications, and sexually transmitted infections (World Health Organization, 2024b).

A study conducted in Morocco among migrant women, most of whom had migrated from Sub-Saharan Africa, found that 30% of participants (*n* = 151) had experienced female genital mutilation, and 76.2% reported experiencing SGBV during migration (Acharai et al. 2023). In Finland, a study revealed high rates of PTEs, such as physical harm and sexual violence, among Somali (69%) and Kurdish (72%) women in their countries before migration (Castaneda et al. 2017). A study conducted in Serbia included 922 migrants. Among them 302 were women, mainly from Syria and Afghanistan, including those in vulnerable situations, such as those who were pregnant (18%). Over one-quarter of all participants had experienced violent events during their journey, and 11% of the female victims had sustained physical injuries. The most common mental health conditions were anxiety and adjustment disorders. (Arsenijević et al. 2017.)

Compared to women in the general population, women from ethnic minority backgrounds are at a higher risk of developing mental health conditions such as post-traumatic stress disorder (PTSD), partly due to greater exposure to intimate forms of violence, including sexual abuse (Knipscheer et al. 2020; Vallejo-Martín et al. 2021). A Finnish study found that 89% of asylum seekers (aged ≥ 18 years) had experienced at least one PTE before arriving in Finland. Half were assessed as being at medium or high risk for psychological trauma, with women, particularly those from African countries other than North Africa, more likely than men to belong to the high-risk group. Women more frequently reported experiences of sexual violence than men. (Garoff et al. 2021.)

Some previous studies have examined how trauma and psychological symptoms influence both maternal and child outcomes during and after pregnancy. Traumatic events in a woman’s life, such as experiences of violence and persecution, can have profound and long-lasting effects on both physical and psychological well-being, significantly hindering their ability to care for their children, if they have any, and to integrate into the host country. (Davaki 2021; Jarallah and Baxter 2019; Swatzyna and Pillai 2013.) Studies on the mental well-being of women in vulnerable situations have highlighted the impact of PTEs on pregnancy, childbirth, and the postpartum period (e.g., lower birth weight and premature birth) (Torche 2011). A review study indicated that women with prior trauma exposure are more susceptible to PTSD symptoms, particularly when facing pregnancy-related stressors such as miscarriage or physical and emotional changes associated with pregnancy (Born et al. 2005). Another study showed that pregnant women with a history of PTEs were more likely to experience high-risk pregnancies, birth complications, and postpartum PTSD compared to women without such experiences (Lev-Wiesel et al., 2009). Additionally, asylum-seeking women exhibit higher rates of mental health conditions (including postnatal and postpartum depression and PTSD) than women in the host country (Heslehurst et al. 2018).

However, a significant research gap persists regarding the psychological impact of PTEs, particularly sexual violence, on asylum-seeking women of reproductive age, especially those with children. Further research is needed to examine background factors that may influence women’s mental health. This knowledge is also essential from the perspective of parental resources, which have thus far received relatively little attention in research. This study utilizes data from the Asylum Seekers Health and Wellbeing Survey (TERTTU) conducted in Finland in 2018 (Skogberg et al. 2019). In 2018, a total of 4,548 individuals applied for asylum in Finland (Finnish Immigration Service, 2019b). Of these, 2,409 were first-time applicants and 30% were women (Finnish Immigration Service, 2018). Most asylum seekers originated from Iraq, Russia, Turkey, Iran, Somalia, Syria, Afghanistan, and Nigeria. Most of them had fled war or persecution, often due to factors such as religion or sexual and gender identity (Finnish Immigration Service, 2018; Ministry of the Interior, 2019).

This study aims to examine PTEs and current mental health symptoms and symptoms indicating psychological trauma among asylum-seeking women of reproductive age. The study focuses particularly on sexual violence, as more information is needed on its impact on women’s mental health. The study explores contextual risk factors (such as pre-migration PTEs and socioeconomic circumstances) that may contribute to these mental health conditions.

## Material and methods

### Study population

The Asylum Seekers Health and Wellbeing Survey (TERTTU) was coordinated by the Finnish Institute for Health and Welfare (Skogberg et al. 2019) and conducted in collaboration with the Finnish Immigration Service and the reception centres. The TERTTU Survey invited all newly arrived asylum seekers who had applied for asylum in Finland between February 19 and December 31, 2018, to participate. During the data collection, Finnish Immigration Service provided register data on all newly arrived asylum seekers in Finland. Data on gender, nationality and age were transferred electronically to the research team weekly. Participants were recruited approximately two weeks after submitting their asylum application.

**Fig. 1:**
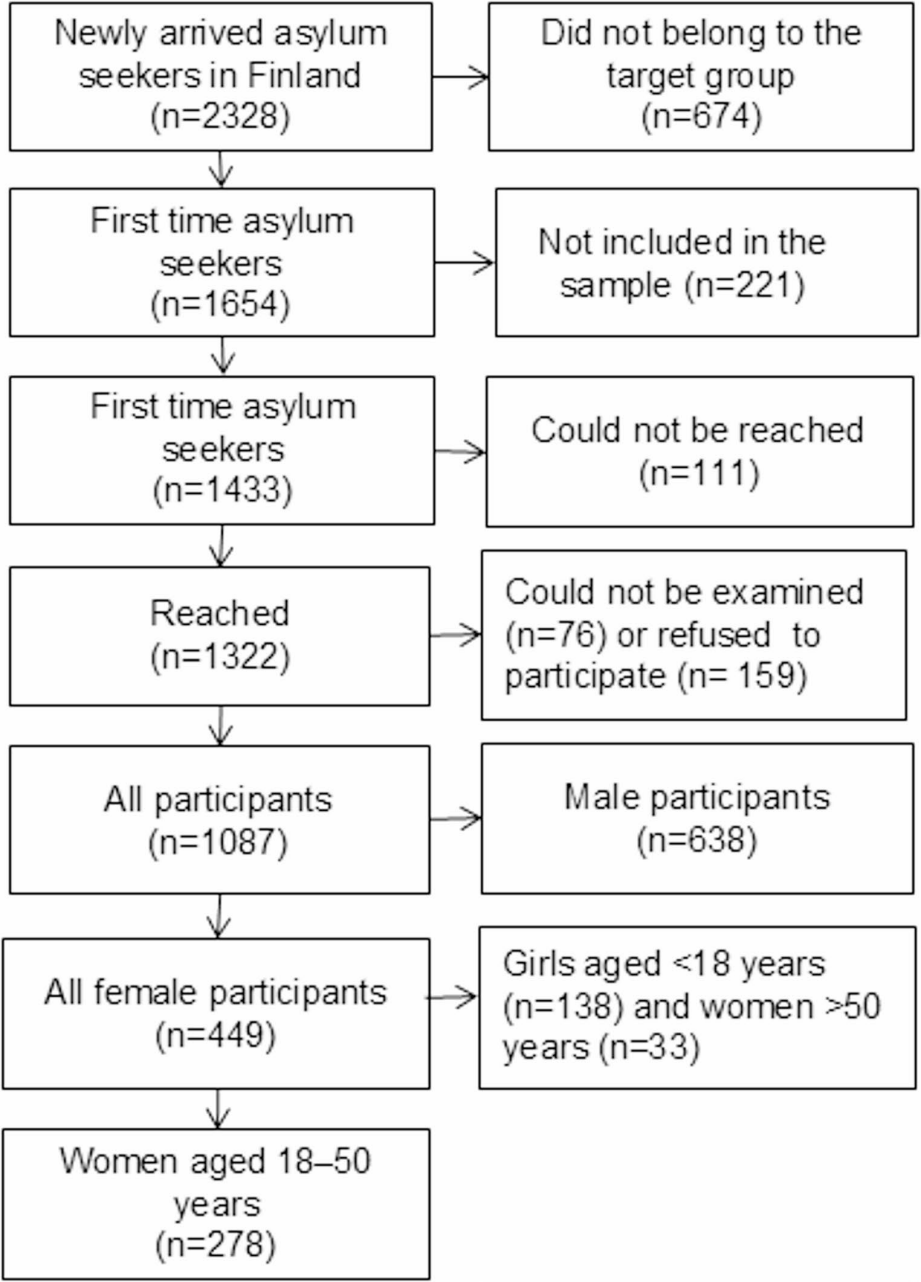
Flow chart of the selection of the study participants

In total, 2,328 newly arrived asylum seekers were registered in Finland during the study period (Fig. 1). Of them, 674 fulfilled at least one of the exclusion criteria: residence in a detention centre; previous application for asylum in another country and transfer to Finland based on international agreements (EU internal transfer); having been returned to Finland according to the EU Dublin Regulation; previous application for residence permit in Finland or health reasons reported by reception centre staff preventing participation in the study. Additionally, 221 persons were not contacted for technical reasons, resulting in a study sample of 1,433 participants. A total of 1,087 individuals participated in the survey, of whom 449 were women. The participation rate among invited women was 75.8%. In this study, women of reproductive age (aged 18–50 years, *n* = 278) were included.

Participants were grouped into four regions of origin based on their country of birth: (1) Russia and the former Soviet Union (FSU), (2) the Middle East and North Africa (MENA), (3) the rest of Africa (later referred to as Africa), and (4) Other countries. The MENA group included mainly citizens of Turkey, Iran, and Iraq, while the Africa group included mainly citizens of Somalia, Nigeria, Angola, and Cameroon. The countries of birth in the Other countries group included, for example, Nicaragua, Albania, Bangladesh, India, Cuba, Kosovo and Sri Lanka. Due to the small size of the Other countries group, it was combined with the Russian and FSU group in the model selection and logistic regression analyses.

### Data collection

The data collection for the TERTTU Survey was carried out by eight trained research nurses with multilingual and multiethnic backgrounds, who also contributed to planning the survey and interpreting the results. The survey included a face-to-face interview and health examination. Whenever possible, the women were interviewed by a female research nurse. All except one of the nurses had a degree in healthcare. The research nurses spoke the most commonly used languages among the participants, and each interview was conducted either in the participant’s first language or in a language both parties could speak fluently enough (Skogberg et al. 2019). When needed, a professional interpreter was used during interviews. A total of 39% of the participants required interpretation, and most of these interviews were conducted by telephone contact, which helped preserve participant anonymity.

The survey materials provided to participants (including information leaflets, consent forms, and questionnaires) were translated into the most common languages spoken among asylum seekers arriving in early 2018: English, Arabic, Somali, Persian, the Sorani dialect of Kurdish, and Russian. The structured interview included questions on participants’ background, health, and well-being, covering topics such as asylum-seeking journey, socio-economic status, health problems and symptoms, PTEs, mental health conditions, and SRH. Most participants responded through face-to-face interviews. During the interview, after the questions concerning PTEs, adults were asked to self-complete the Process of Recognition and Orientation of Torture Victims in European Countries to Facilitate Care and Treatment (PROTECT) questionnaire and the Hopkins Symptom Checklist (HSCL-25).

In addition, completing the structured questionnaire independently was deemed the most practical approach for collecting information on SRH. If needed, participants could receive assistance from a research nurse. In total, 37% of those offered the self-administered questionnaire required help, often due to limited reading or writing skills. A more detailed description of survey methods has been provided elsewhere (Skogberg et al. 2019).

## Variables

### Background information

Information on sex, age, and country of birth was obtained from the electronic database maintained by the Finnish Immigration Services. Sex was categorized as either male or female. Age was calculated using the year of birth and the time of the study, and categorized into two groups: 18–29 years and 30–50 years. Other background variables (education, language and reading skills, family situation, pregnancy status, and births) were based on self-reported information, with classification details provided in Appendix 1.

### Trauma

PTEs before arriving in Finland were assessed using questions adapted from the Harvard Trauma Questionnaire (HTQ, 18) (Mollica et al., 1992). The questionnaire included items on various types of PTEs: combat situations, natural disasters, witnessing violent injury or death, being physically harmed, experiencing severe physical violence, imprisonment or kidnapping, torture, sexual violence, and being forced or cheated into doing something against one’s will. Each item had response options of “yes” and “no”. In this study, two binary items were created to indicate: (1) exposure to sexual violence, and (2) exposure to other PTEs. Both items were coded as “yes” or “no”.

### Psychological symptoms

HSCL-25 was used to detect depressive and anxiety symptoms. It is a validated 25-item self-report tool, widely used for trauma-affected people (Derogatis et al., 1974). The scale has 10 items for anxiety and 15 for depression. Each item is rated from 1 (“not at all”) to 4 (“often”). HSCL mean scores (ranging from 1.0 to 4.0) were calculated for each participant. The recommended cut-off of 1.75 was used in logistic regression analyses to indicate clinically significant symptoms (Nettelbladt et al. 1993; Winokur et al. 1984).

The PROTECT Questionnaire facilitates early recognition of individuals who may have experienced traumatic events. These can include torture or psychological, physical, or sexual violence. The questionnaire assesses symptoms that indicate the likelihood of such experiences and identifies those who may need support. (Boillant et al. 2012.) It includes PTSD symptoms (4 items: nightmares, anger, thinking about painful past events, feeling scared or frightened), symptoms found in both major depressive disorder and PTSD (4 items: problems falling asleep, forgetting things, losing interest in things, trouble concentrating), and pain symptoms (2 items: headaches, other physical pains). The answer options are “yes” or “no.” The total number of “yes” responses is summed to indicate symptom level: low (0–3), medium (4–7), and high (8–10). For logistic regression analyses, the medium- and high-symptom categories are combined into a single category. For clarity, this study refers to these results as “symptoms indicating psychological trauma.”

The items used in this study are described in more detail in the Supplementary material (Table S1).

### Statistical analyses

All analyses in this study were performed using SAS statistical software, version 9.4. Analysis weights were used to reduce nonresponse bias. The weights were calculated using the inverse probability method. A more detailed description of this method has been provided elsewhere (Majlander et al. 2025). The weights were used in the analyses in which prevalences of PTEs, depressive symptoms, anxiety symptoms, and symptoms indicating psychological trauma were examined. For each outcome, weighted frequencies and their 95% confidence intervals (CI) were calculated for both binary and multinomial variables. The confidence intervals were calculated using a logit transformation.

Logistic regression analysis was conducted to identify the best models for depressive and anxiety symptoms (HSCL ≥ 1.75) and symptoms indicating psychological trauma (PROTECT medium or high) (Burnham and Anderson 2002). Model selection was based on the Akaike Information Criterion (AIC), as it was considered the most appropriate method for identifying factors that increase the risk of mental health symptoms and symptoms indicating psychological trauma. In the preliminary analyses, each potential variable (e.g., language proficiency, family situation, reproductive health events) was examined individually to ensure that the number of cases was sufficient for inclusion in the model. Eleven variables were selected based on their relevance to the research question, specifically in identifying mental health problems among female asylum seekers. The variables included sexual violence, other PTEs, births, pregnancy status, family situation, age, education, spouse, language skills, reading skills, and country group. To perform AIC analyses, the Other countries group was merged with Russia and the FSU group. The AIC values were calculated for all possible combinations (*n* = 2,047). The best 16 models are presented in Supplementary material Tables 2 and 4. The models with the variables that best explained having depressive and anxiety symptoms and symptoms indicating psychological trauma were selected, indicating the optimal balance between explanatory power and model simplicity. In addition, Akaike weights were calculated to assess the importance of each variable (Supplementary material Tables 3 and 5). The model with the lowest AIC value, in which all variables had an Akaike weight greater than 0.5, was selected as the best-fitting model. These weights supported the selection of the final models. Statistical significance was assessed using the p-value from the F-test (Pr > F). Results are presented as odds ratios (ORs), 95% CIs, and p-values for the final models.

## Results

Most of the women were aged 30–50 years, but nearly half (47%) of the women in the Africa group were aged 18–29 years (Table 1). In the Africa group, 56% had no family in Finland, and 75% had no spouse. Among women in Russia and FSU, and the MENA group, 20–23% had three or more births, and 25% of the women in the Africa group were pregnant at the time of the survey. There were differences in educational background: 45% of women in the Africa group and 21% in the MENA group had no formal education or had only completed primary school. Language skills also varied, with 32% of women in the Africa group and 34% in the Russia and FSU group speaking only their first language. Additionally, 32% of women in the Africa group could read only simple texts.

**Table 1:** Background of the study participants, weighted percentages (95% confidence intervals)

	Russia and the former Soviet Union *n* = 85 % (95% Cl)	Middle East and North Africa *n* = 119 % (95% Cl)	Africa *n* = 47 % (95% Cl)	Other countries *n* = 27 % (95% Cl)
Age				
18–29 years	29.3 (20.7–39.8)	31.0 (23.2–40.0)	46.5 (32.3–61.2)	27.6 (12.1–51.4)
30–50 years	70.7 (60.2–79.3)	69.0 (60.0–76.8)	53.5 (38.8–67.7)	72.4 (48.6–87.9)
Family ^a^				
Children and/or spouse in Finland	88.3 (79.6–93.6)	75.2 (66.2–82.5)	43.9 (29.8–59.2)	56.9 (36.4–75.2)
No family in Finland	11.7 (6.4–20.4)	24.8 (17.5–33.8)	56.1 (40.8–70.2)	43.1 (24.8–63.6)
Spouse				
Spouse in Finland or abroad	81.1 (71.4–88.0)	75.9 (66.6–83.2)	25.2 (13.7–41.7)	50.6 (31.4–69.7)
No spouse/spouse dead	18.9 (12.0–28.6)	24.1 (16.8–33.4)	74.8 (58.3–86.3)	49.4 (30.3–68.6)
Pregnancy				
Pregnant at the time of the study	6.3 (2.7–14.2)	7.5 (3.7–14.6)	24.7 (14.3–39.2)	NA ^b^
Births				
No births	29.9 (20.9–40.7)	31.5 (23.2–41.0)	48.1 (33.2–63.4)	48.7 (29.3–68.5)
1–2	47.5 (36.9–58.4)	48.8 (39.4–58.2)	39.0 (25.4–54.6)	41.7 (24.3–61.4)
≥ 3	22.6 (14.7–33.0)	19.8 (13.2–28.5)	12.8 (5.1–28.7)	NA ^a^
Education				
No education/only elementary school	NA ^b^	21.2 (14.6–29.8)	45.4 (31.2–60.4)	NA ^a^
High school/vocational training/University degree	95.1 (87.8–98.2)	78.8 (70.2–85.4)	54.6 (39.6–68.8)	83.5 (61.8–94.1)
Language skills				
First language only	34.0 (24.6–44.7)	27.4 (20.1–36.1)	31.9 (19.4–47.7)	22.7 (10.0–43.6)
First and other languages	66.0 (55.3–75.4)	72.6 (63.9–79.9)	68.1 (52.3–80.6)	77.3 (56.4–90.0)
Reading skills				
All texts	97.7 (91.1–99.4)	87.5 (80.1–92.4)	67.7 (53.3–79.4)	86.7 (64.0–96.0)
Simple texts only	NA ^b^	12.5 (7.6–19.9)	32.3 (20.6–46.7)	NA ^b^

a spouse or children < 18 years

b NA = Not available as too few observations (n = < 5) for statistical analysis

All groups reported PTEs, but women in the Africa group stood out (Table 2). Among them, 44% had experienced combat situations, 70% had witnessed violent injury or death, 73–80% had experienced physical harm or severe physical violence, 44% had been imprisoned or kidnapped, 53% had been tortured, 58% had experienced sexual violence, and 72% had been forced or cheated.

**Table 2:** Potentially traumatic experiences and mental health symptoms in asylum-seeking women aged 18‒50 years, weighted percentages (95% confidence intervals)

PTE	Russia and the former Soviet Union *n* = 85 % (95% Cl)	Middle East and North Africa *n* = 119 % (95% Cl)		Africa *n* = 47 % (95% Cl)	Other countries *n* = 27 % (95% Cl)	*p*–value ^a^
Combat situation	14.1 (8.2–23.1)		28.1 (20.3–37.4)	44.4 (30.5–59.3)	39.1 (21.8–59.7)	0.003
Natural disaster	11.7 (6.4–20.4)		18.1 (12.3–25.9)	21.6 (11.6–36.6)	32.0 (16.7–52.4)	0.119
Seeing violent injury or death	34.2 (24.9–44.8)		32.3 (24.2–41.6)	70.0 (54.7–81.8)	54.7 (34.9–73.1)	< 0.001
Physical harm	54.3 (43.7–64.5)		31.5 (23.6–40.7)	72.6 (56.2–84.6)	42.2 (24.3–62.4)	< 0.001
Severe physical violence	33.2 (24.0–43.8)		23.4 (16.5–32.1)	79.7 (64.5–89.5)	39.1 (21.8–59.7)	< 0.001
Imprisoned or kidnapped	11.7 (6.4–20.4)		20.3 (13.7–29.0)	44.1 (29.9–59.3)	NA ^b^	NA ^b^
Tortured	8.2 (4.0–16.2)		28.9 (21.3–38.0)	52.5 (37.6–67.0)	36.9 (19.5–58.6)	< 0.001
Sexual violence	16.6 (10.1–26.0)		16.7 (11.1–24.3)	57.6 (42.3–71.7)	22.7 (10.0–43.6)	< 0.001
Forced or cheated	28.3 (19.8–38.7)		29.1 (21.5–38.1)	71.5 (56.0–83.1)	25.8 (12.2–46.5)	< 0.001
ANY PTE	75.4 (65.2–83.3)		66.4 (57.2–74.5)	89.5 (73.7–96.3)	81.4 (63.5–91.6)	0.025
HSCL						
Depression	39.6 (29.8–50.4)		45.4 (36.3–54.9)	70.9 (55.2–82.8)	42.2 (24.3–62.4)	0.011
Anxiety	42.2 (32.1–53.0)		38.2 (29.5–47.7)	52.0 (36.7–66.9)	42.2 (24.3–62.4)	0.517
HSCL mean score	40.8 (30.9–51.6)		44.6 (35.5–54.1)	62.1 (46.1–75.9)	42.2 (24.3–62.4)	0.161
PROTECT						
Low	54.3 (43.7–64.5)		37.5 (29.1–46.7)	33.8 (21.3–49.1)	44.4 (26.4–64.0)	0.130
Medium	34.0 (24.8–44.6)		39.1 (30.5–48.4)	35.7 (22.9–51.0)	40.0 (22.1–61.0)	
High	11.7 (6.4–20.4)		23.4 (16.4–32.3)	30.5 (18.1–46.5)	15.5 (6.4–32.9)	
Medium or high	45.7 (35.5–56.3)		62.5 (53.3–70.9)	66.2 (50.9–78.7)	55.6 (36.0–73.6)	0.087

a P-value of chi-square statistic for difference between all groups

b NA = Not available as too few observations (n = < 5) for statistical analysis

Among women from the Africa group, 71% reported depressive symptoms, while 42–45% of women from the MENA and Other countries groups reported similar symptoms. Over half (52%) of women from the Africa group reported anxiety symptoms, as did 42% of women from the Russia and FSU and Other countries groups. In the Africa group 66% reported symptoms indicating psychological trauma (medium or high), followed by the MENA group (63%), the Other countries group (56%), and the Russia and FSU group (46%).

Logistic regression analysis, guided by AIC-based model selection, identified that sexual violence was most strongly associated with depressive and anxiety symptoms (HSCL ≥ 1.75) among asylum-seeking women (Table 3). Women who had experienced sexual violence had over six times higher odds of having these symptoms (OR = 6.33, 95% CI: 2.86–14.05) compared to those who had not. Exposure to other PTEs than sexual violence also showed elevated odds (OR = 2.00, 95% CI: 0.98–4.09), although the association was non-significant (*p* = 0.059). Family situation was also associated with depressive and anxiety symptoms: women without family in Finland had nearly twice the odds of depressive and anxiety symptoms (OR = 1.98, 95% CI: 0.99–3.97) compared to those with family present. Finally, age was a significant factor: younger women (aged 18–29 years) had twice the odds of experiencing depressive and anxiety symptoms (OR = 2.07, 95% CI: 1.10–3.89) compared to women aged 30–50 years.

**Table 3:** Final weighted logistic regression model for depressive and anxiety symptoms (HSCL) among asylum-seeking women aged 18‒50 years

Variable ^a^	OR (95% Cl) ^b^	*p*-value
No sexual violence	1	< 0.0001
Sexual violence	6.33 (2.86–14.05)	
No PTE	1	0.059
Other PTE	2.00 (0.98–4.09)	
Family in Finland	1	0.054
No family	1.98 (0.99–3.97)	
Age 30–50	1	0.025
Age 18–29	2.07 (1.10–3.89)	

a Variables were selected to the model based on the Akaike Information Criterion

b Adjusted odds ratio (OR) and 95% confidence intervals (CI)

Logistic regression analysis, guided by AIC-based model selection, identified sexual violence (OR = 4.71, 95% CI: 1.99–11.17) and other PTEs (OR = 3.13, 95% CI: 1.46–6.73) as significantly associated with symptoms indicating psychological trauma (PROTECT medium or high) among asylum-seeking women (Table 4). Parity showed a U-shaped association: both women with no births (OR = 2.22, 95% CI: 1.12–4.41) and those with three or more births (OR = 3.62, 95% CI: 1.43–9.14) had elevated odds of symptoms indicating psychological trauma compared to those with 1–2 births. Language skills were also associated: multilingual women had higher odds (OR = 2.37, 95% CI: 1.23–4.55) compared to those who spoke only their first language. Women from the MENA group had higher odds (OR = 2.42, 95% CI: 1.19–4.95) compared to women from Russia and FSU, as well as those in the Other countries group. Pregnancy status, age, and reading skills were included in the model, but none showed a statistically significant association.

**Table 4:** Final weighted logistic regression model for symptoms indicating psychological trauma (PROTECT) among asylum-seeking women aged 18‒50 years

Variable ^a^	OR (95% Cl) ^b^	*p*-value
No sexual violence	1	0.001
Sexual violence	4.71 (1.99–11.17)	
No PTE	1	0.004
Other PTE	3.13 (1.46–6.73)	
No births	2.22 (1.12–4.41)	0.009
1–2 births	1	
≥ 3 births	3.62 (1.43–9.14)	
Pregnant	1	0.073
Not pregnant	2.89 (0.90–9.21)	
Age 18–29 years	1.82 (0.91–3.63)	0.090
Age 30–50 years	1	
First language only	1	0.010
First and other languages	2.37 (1.23–4.55)	
Reads all kind of texts	1	0.178
Reads only simple texts	1.93 (0.74–5.00)	
Russia and FSU and Other countries	1	0.050
Middle East and North Africa	2.42 (1.19–4.95)	
Africa	1.22 (0.49–3.08)	

a Variables were selected to the model based on the Akaike Information Criterion

b Adjusted odds ratio (OR) and 95% confidence intervals (CI)

## Discussion

In this study, women seeking asylum, particularly those from Africa, were found to have experienced PTEs that should be considered in service provision. Depressive symptoms and anxiety symptoms were common among asylum-seeking women, and the experiences of PTEs were associated with increased odds of such symptoms. Key findings from the final models of depressive and anxiety symptoms and symptoms indicating psychological trauma identified the severe impact of sexual violence on women’s mental health. These findings are consistent with previous research (Cignacco et al. 2018; Knipscheer et al. 2020; Kurth et al. 2010; Vallejo-Martín et al. 2021), which further highlights the vulnerability of asylum-seeking women. Studies have shown that they experience high rates of SGBV while fleeing to EU countries, including forced transactional sex, coercion by smugglers, and mistreatment by coastguards (Freedman 2016).

Other factors were also found to be associated with depressive and anxiety symptoms. These included other PTEs, absence of family, and younger age. Furthermore, parity (no births and ≥ 3 births), country group (MENA group), and, somewhat unexpectedly, multilingualism were found to be associated with symptoms indicating psychological trauma. In the AIC-guided model analysis, pregnancy status, age, and reading skills were identified as potential risk factors, although none showed a statistically significant association with the symptoms. These findings reflect the complexity of mental health shaped by individual and situational factors (Fried and Robinaugh 2020). While supported by previous research, further studies are needed due to the multifaceted nature of mental health. Exploring complex issues requires access to large, high-quality datasets and longitudinal research designs.

PTEs can have profound and lasting effects on women’s physical and psychological well-being (Cayreyre et al. 2024). A register-based study conducted in Finland found that female immigrants from the MENA and Eastern Europe had a significantly higher incidence of PTSD compared to people born in Finland, likely due to migration from conflict-affected regions (Markkula et al. 2017). Similarly, among Ukrainian refugees in the Czech Republic, women had 1.77 times greater odds of experiencing depressive and anxiety symptoms compared to men (Guerrero et al. 2023). Asylum-seeking women with experiences of PTEs face numerous psychosocial stressors related to their life circumstances. These include forced migration, challenging living conditions in the host country, and uncertainty about the future. (Kurth et al. 2010; Poya 2021.) Feelings of insecurity and isolation, often exacerbated by language barriers, along with homesickness, family separation, financial concerns, and experiences of hostility and discrimination in the host country, tend to intensify during the asylum process (Zecchinato et al. 2025).

The reasons why women without children and those with three or more children had higher odds of symptoms indicating psychological trauma compared to those with one or two children remain unclear. However, previous research suggests that pregnant asylum-seeking women and single mothers with young children are particularly vulnerable, facing increased stress due to linguistic, cultural, social, and financial challenges in a new environment (Gewalt et al. 2018; Stewart et al. 2015). In addition, legal uncertainties and fear of deportation can further harm the mental and physical health of these women (Gewalt et al. 2018). However, findings from this and previous studies indicate that children can serve as a source of strength and motivation, helping women cope with complex and challenging situations (Holmyard et al. 2023). Importantly, women are not merely victims; they also demonstrate resilience and the ability to survive in adversity. While traumatic experiences increase the risk of mental health problems, factors such as social support, access to services, and as shown also in the present study, the presence of family can strengthen resilience, especially among pregnant asylum-seeking women and mothers (Zecchinato et al. 2025).

The impact of trauma on women who are pregnant and/or have children should be broadly acknowledged and considered in the SRH services. Pregnant women and mothers who have experienced PTEs should be considered in health care revisions to ensure that they receive appropriate support and trauma‑informed care. Several research findings show that when a woman experiences psychological distress during pregnancy, such as depression, anxiety, or stress, it may affect not only her own well-being but also the development of the mother-infant relationship, including the quality of early interaction and bonding (Iliadou et al. 2019; Staneva et al. 2015). The vulnerable situation of asylum-seeking women with children calls for special attention within social and healthcare services to ensure that their needs are identified and addressed (Rowe et al. 2023). Due to global conflicts, this issue increasingly affects European countries. Women arriving from conflict-affected regions will likely continue to seek asylum in Finland in the future.

The reason why multilingual women had higher odds of symptoms indicating psychological trauma compared to those who spoke only their mother tongue remains unclear. One possible explanation is that many of the women in this study originated from countries (particularly in the MENA and Africa group), where multilingualism is common (Heugh 2019; Zakharia 2016).

In summary, the findings of this and previous studies demonstrate that traumatic experiences and migration-related stress have a significant impact on women’s lives in various ways (Davaki 2021; Hedström and Herder 2023; Jarallah and Baxter 2019). These impacts should be more thoroughly acknowledged in both research and service systems. In addition, actions addressing SGBV must include both support for victims and accountability for perpetrators, particularly in conflict situations, as sexual violence is a deliberate weapon of war.

The sensitivity of this issue must be acknowledged, as questions related to violence, PTEs, and mental health are often delicate. Many women choose to conceal experiences of violence (Aktar et al. 2020) due to cultural taboos, stigma, fear of discrimination, distrust in authorities, concerns about migration status, or the emotional burden of disclosure. As a result, under-reporting remains a major barrier to addressing PTEs, including SGBV (Jolof et al. 2024; Tan and Kuschminder 2022). In the present study, response bias related to questions about PTEs and mental health symptoms was not formally assessed. However, such bias may have influenced the results, as a separate study involving interviews with TERTTU research nurses indicated that some participants found the questions challenging (Ahmed Haji Omar A., 2019). Also previous studies show that many women in vulnerable situations choose not to disclose experiences of violence or trauma due to stigma, fear, and other barriers, making under-reporting a major challenge in addressing SGBV (Ahmed et al. 2020; Jolof et al. 2024; Tan and Kuschminder 2022).

The findings of this study underscore the vulnerability of asylum-seeking women arriving in Finland. Comprehensive health examinations should be provided shortly after arrival. Healthcare services must be prepared to identify women requiring special support, including victims of human trafficking or sexual exploitation (see also Bakken et al. 2015; Glover 2014).

The outcomes of this study are influenced by multiple interconnected factors, which makes them inherently complex to examine; however, this study has several strengths. This study draws on a uniquely large sample of recently arrived asylum-seeking women in Finland. However, after restricting the data for this study to women of reproductive age by country groups, the sample becomes small enough that not all analyses are feasible.

The sensitivity of the topic and the reliability of the data were carefully considered throughout the TERTTU Survey. The participants were explicitly informed about personal data protection, the independence of the study from health and social services and social security, as well as its separation from the asylum-seeking process (Skogberg et al. 2019). Furthermore, the survey materials were translated into additional languages as needed. Standardized procedures were used in interviews with asylum-seeking women. The nurses received training on ethical issues, research themes, and how to ask sensitive questions. In most cases, the nurse and participant shared a common language and gender, which likely enhanced trust and communication. Based on the information provided by the research nurses, no gaps or frequently missing data were observed in the questionnaire responses, including those containing the HSCL and PROTECT instruments, which are designed to assess mental health symptoms and symptoms indicating psychological trauma (Ahmed Haji Omar 2019).

However, the study also has limitations. The size of the dataset limited the ability to examine interaction effects or other main effects, such as the impact of health and functional ability on mental health. For the model selection analyses, the Other countries group and the Russian and FSU group had to be combined. This may have affected the results, as women in these groups differed in many contextual factors (e.g., family situation), even though they had some similarities (e.g., age). In addition, a clear limitation of the study is that the perpetrator was not specified in the questions related to violence, due to the aim of keeping the questionnaires concise, as they covered several themes. Generalizing the findings on the prevalence of mental health symptoms and symptoms indicating psychological trauma and their contextual risk factors should be done with caution, as the countries of origin and reasons for seeking asylum vary from year to year.

As the results of this study show, both mental health, trauma, and contextual factors in the women’s lives are complex and warrant further research. It was not possible to analyze how the number and/or the age of children relate to depressive and anxiety symptoms and symptoms indicating psychological trauma. Future studies should specifically examine the resources and stressors experienced by asylum-seeking women with a history of trauma to better understand the mechanisms influencing their mental health. For instance, questionnaires could also include items on factors supporting well-being, as religion and faith, which may serve as important coping mechanisms (Gewalt et al. 2018).

Qualitative research is essential for understanding lived experiences and contextual factors often overlooked by quantitative methods, such as women’s individual circumstances, care needs, and coping strategies. Moreover, undocumented individuals, excluded from the TERTTU Survey, represent a highly vulnerable group requiring targeted research. Finally, studies on healthcare professionals could help identify gaps in trauma-informed care competencies.

## Conclusion

This study sought to advance understanding of the mental health conditions encountered by asylum-seeking women, who frequently face structural and social marginalization. Many of these women are of reproductive age and either have children or are likely to have children in the future. The effects of trauma and stress are reflected not only during pregnancy, childbirth, and the postpartum period, but also later in parenting, and may indirectly affect their children as well (Kaitz et al. 2009; Thomson 2013). This study identified PTEs and contextual risk factors associated with mental health symptoms and symptoms indicating psychological trauma. Given the high prevalence of trauma, there is an urgent need for the universal provision of culturally competent, trauma‑informed care, rather than limiting such care only to those with a documented or repeated history of trauma (Donnelly et al. 2023; Fair et al. 2020). Women who have experienced sexual violence represent a high-risk group.

## Supplementary Information

Below is the link to the electronic supplementary material.

Supplementary Material 1 Table S 1 Variables (DOCX 18.8 KB)

Supplementary Material 2 Table S 2–5 AIC-based model selection for mental health symptoms (DOCX 19.1 KB)

## Data Availability

The data that support the findings of this study can be obtained from THL but restrictions apply due to data protection regulations, ethics approval of the surveys and the subjects` consent to participate. Thus the data are not publicly available.
