# Supplementary material for: Potentially traumatic experiences and mental health among asylum-seeking women of reproductive age: The significance of sexual violence and contextual risk factors
Source: Arch Womens Ment Health. 2026 Apr 21;29(3):66. doi: 10.1007/s00737-026-01697-z (PMC13099702; doi:10.1007/s00737-026-01697-z)
Supplement: Supplementary file 1 — Supplementary Material 1 Table S 1 Variables (DOCX 18.8 KB) [file 737_2026_1697_MOESM1_ESM.docx]

**Table S 1**

Variables

| **Background information** | | | |  |
| --- | --- | --- | --- | --- |
| **Theme** | **Question / Data source** | **Answer options** | **Categories** |  |
| Gender | Register information | – | –Man –woman |  |
| Country of birth | Register information | – | –Russia or the former Soviet Union  –the Middle East and North Africa –Other African countries (exl. North Africa)  –Other countries (the most common countries of birth were Nicaragua, Albania, Bangladesh, India, Cuba, Kosovo, and Sri Lanka).  Combined: – Russia or the former Soviet Union and other countries (reference group)  –The Middle East and North Africa  –Africa |  |
| Age | Register information | – | 18–29, 30–35 |  |
| Education | What is your highest completed education or degree? | “Unknown”, “No education”, “Basic education 3 years or less”, “Basic education 4 to 6 years”, “Basic education 7 to 9 years”, “Upper secondary education / High school”, “Vocational school”, “Tertiary education” [bachelor’s or master’s degree or higher] | –No education or only elementary school education  –High school, vocational training or university degree  (reference group) |  |
| Reading skills | How well can you read? | “I can read all kinds of text”, “I can read simple text”, “I can read names, words and very simple sentences”, “I can not read at all” | –Can at most read simple texts  –Can read all kind of texts (reference group) |  |
| Writing skills | How well can you write? | “I can write all kinds of text “, “I can write simple text”, “I can write familiar names, words and very simple sentences”, “I can not write at all” | –Can at most write simple texts  –Can write all kind of texts (reference group) |  |
| Language skills | Do you speak any other languages besides your mother tongue? | “No”, “Arabic”, “Kurdish”, ” Dari”, “Persian”, “Somali”, “Russian”, “English”, “French”, “Other language, what?” | –Speaks only her mother tongue (reference group)  –Speaks several languages |  |
| Family | Do you have any of the following living family members: a spouse, children under 18 years, children over 18 years, siblings, father, mother. | “No”, “Dead”, “In Finland”, “Elsewhere, in [country/countries]” | –No spouse  –Spouse in Finland or abroad Combined: –Family (spouse or/and children) in Finland (reference group) –No family at all |  |
| **Reproductive health** |  |  |  |  |
| **Theme** | **Question** | **Answer options** | **Categories** |  |
| Pregnancy | Are you pregnant? [If the woman answered yes, pregnancy weeks were either recorded or marked unknown. When necessary, the research nurses were instructed to help to estimate the pregnancy weeks.] | “Yes”, “No”, “I do not know” | –Yes (reference group) –No |  |
| Previous births | How many births have you had? | –The number was recorded (0 if none) | 0, 1–2, 3 or more |  |
| Potentially traumatic experiences | | | |  |
| Theme | | Question | Answer options | Categories |
| Harvard Trauma Questionnaire (HTQ, 18) | Have you experienced any of the following distressing events:  1) Experienced a combat situation in a war? 2) Been the victim of a natural disaster? 3) Witnessed violent injury or death? 4) Been the target of physical harm? 5) Been a target of severe physical violence? 6) Been imprisoned or kidnapped? 7) Been tortured? 8) Experienced sexual violence? 9) Been forced or cheated into doing something that you did not want to do?  [Answer options: no; yes, before the asylum–seeking journey; yes, during the asylum–seeking journey] | “Yes” and “No” | –Sexual violence  yes  no (reference group) –Other potentially traumatic experiences  yes  no (reference group) |  |
| **Mental health** |  |  |  |  |
| **Theme** | **Question** | **Answer options** | **Categories** |  |
| The Hopkins Symptom Checklist–25 (HSCL–25) | 10–item subscale for anxiety and a 15–item subscale for depression | Each item scored in terms of frequency from 1 (not at all) to 4 (often). HSCL mean scores (ranging from 1.0–4.0) are calculated for each case. | cutoff of 1.75 indicating significant depressive and anxiety symptom  <1.75 (reference group) |  |
| The PROTECT Questionnaire (PQ) | Questionnaire include items concerning:  –The frequency of symptoms of PTSD. Four items: nightmares, anger, thinking about painful past events, feeling scared or frightened  –Symptoms of both MDD and PTSD. Four items: problems falling asleep, forgetting things, losing interest in things, trouble concentrating  –Pain symptoms, two items: headaches, other physical pains. | –Yes –No  The number of items for which “yes” is reported is added together to indicate the risk for post-traumatic mental health problems | –Low-risk 0–3 yes answers –Medium-risk 4–7 yes answers –High-risk 8–10 yes answers). Combined:  –Low risk (reference group) –Medium/High Risk |  |
