# Supplementary material for: Potentially traumatic experiences and mental health among asylum-seeking women of reproductive age: The significance of sexual violence and contextual risk factors
Source: Arch Womens Ment Health. 2026 Apr 21;29(3):66. doi: 10.1007/s00737-026-01697-z (PMC13099702; doi:10.1007/s00737-026-01697-z)
Supplement: Supplementary file 2 — Supplementary Material 2 Table S 2–5 AIC-based model selection for mental health symptoms (DOCX 19.1 KB) [file 737_2026_1697_MOESM2_ESM.docx]

**Table S 2**

AIC-based model selection for predicting mental health symptoms among asylum-seeking women aged 18–50 years

| **Model** | **AIC ^a^** |
| --- | --- |
| sexual violence, other PTE, family situation, age, spouse, language skills | 291,397 |
| sexual violence, other PTE, family situation, age, spouse | 291,431 |
| sexual violence, other PTE, family situation, age | **292,103** |
| sexual violence, other PTE, family situation, age language skills | 292,127 |
| sexual violence, other PTE, age, language skills | 292,489 |
| sexual violence, other PTE, pregnancy, family situation, age, spouse, language skills | 292,751 |
| sexual violence, other PTE, pregnancy, family situation, age, spouse | 292,870 |
| sexual violence, other PTE, childbirths, age, language skills | 292,939 |
| sexual violence, other PTE, family situation, age, spouse, reading | 293,075 |
| sexual violence, other PTE, childbirths, family situation, age, spouse, language skills | 293,093 |
| sexual violence, other PTE, age | 293,101 |
| sexual violence, other PTE, family situation, age, reading skills | 293,196 |
| sexual violence, other PTE, family situation, age, education, spouse, language skills | 293,198 |
| sexual violence, other PTE, family situation, age, spouse, language skills, reading skills | 293,271 |
| sexual violence, other PTE, childbirth amount, family situation, age, spouse | 293,383 |
| sexual violence, other PTE, family situation, age, education, spouse | 293,413 |

a AIC = Akaike Information Criterion

**Table S 3**

Relative importance of predictors based on Akaike weights ^a^ in model selection for mental health symptoms among asylum-seeking women aged 18–50 years

| **Variable** | **Sum** |
| --- | --- |
| sexual violence | 0.9999995 |
| age | 0.9158096 |
| other PTE | 0.8428116 |
| family situation | 0.6719453 |
| language skills | 0.5225189 |
| spouse | 0.4488640 |
| births | 0.3473965 |
| pregnant | 0.3316575 |
| reading skills | 0.3222871 |
| education | 0.2877082 |
| country group | 0.1535789 |

a Akaike weights derived from logistic regression models. Higher weights indicate greater relative importance in explaining the outcome.

**Table S 4**

AIC-based model selection for predicting symptoms indicating psychological trauma among asylum-seeking women aged 18–50 years

| **Model** | **AIC ^a^** |
| --- | --- |
| sexual violence, other PTE, childbirths, pregnancy, age, education, language skills, reading skills, country group | 301,027 |
| sexual violence, other PTE, childbirths, pregnancy, age, language skills, reading skills, country group | **301,988** |
| sexual violence, other PTE, childbirths, pregnancy, age, language skills, country group | 302,079 |
| sexual violence, other PTE, childbirths, pregnancy, age, education, spouse, language skills, reading skills, country group | 302,813 |
| sexual violence, other PTE, childbirths, pregnancy, age, education, language skills, reading skills, country group | 302,893 |
| sexual violence, other PTE, childbirths, pregnancy, family situation, age, education, language skills, reading skills, country group | 303,006 |
| sexual violence, other PTE, childbirths, age, education, language skills, reading skills, country group | 303,121 |
| sexual violence, other PTE, childbirths, pregnant, language skills, reading skills, country group | 303,152 |
| sexual violence, other PTE, childbirths, pregnant, language skills, country group | 303,296 |
| sexual violence, other PTE, childbirths, age, language skills, country group | 303,828 |
| sexual violence, other PTE, childbirths, pregnant, age, spouse, language skills, country group | 303,833 |
| sexual violence, other PTE, childbirths, pregnant, age, spouse, language skills, reading skills, country groups | 303,887 |
| sexual violence, other PTE, childbirths, pregnant, age, education, language skills, country group | 303,895 |
| sexual violence, other PTE, childbirths, pregnant, family situation, age, language skills, reading skills, country group | 303,940 |
| sexual violence, other PTE, childbirths, age, language skills, reading skills, country group | 304,050 |
| sexual violence, other PTE, childbirths, pregnant, family situation, age, language skills, country group | 304,051 |

a AIC = Akaike Information Criterion

**Table S 5**

Relative importance of predictors based on Akaike weights ^a^ in model selection for symptoms indicating risk of psychological trauma among asylum-seeking women aged 18–50 years

| Variable | Sum |
| --- | --- |
| sexual violence | 0.9999294 |
| other PTE | 0.9838015 |
| births | 0.9792968 |
| country group | 0.8914174 |
| language skills | 0.8427944 |
| pregnant | 0.7066831 |
| age | 0.6670650 |
| reading skills | 0.6262133 |
| education | 0.4903935 |
| spouse | 0.3121977 |
| family situation | 0.2828109 |

a Akaike weights derived from logistic regression models. Higher weights indicate greater relative importance in explaining the outcome
